# Sleep Disturbances, Fatigue and Immune Markers in the Irritable Bowel Syndrome and Inflammatory Bowel Disease, a Systematic Review

**DOI:** 10.1111/nmo.70133

**Published:** 2025-08-31

**Authors:** Sophie Fowler, Laura R. C. Dowling, Nicole Simm, Nicholas J. Talley, Grace L. Burns, Simon Keely

**Affiliations:** ^1^ School of Biomedical Sciences & Pharmacy, College of Health, Medicine and Wellbeing University of Newcastle New South Wales Australia; ^2^ NHMRC Centre of Research Excellence in Digestive Health University of Newcastle Newcastle New South Wales Australia; ^3^ Immune Health Research Program, Hunter Medical Research Institute New Lambton Heights New South Wales Australia; ^4^ School of Medicine & Public Health, College of Health, Medicine and Wellbeing University of Newcastle New South Wales Australia

**Keywords:** fatigue, inflammation, inflammatory bowel disease, irritable bowel syndrome, sleep

## Abstract

**Background:**

The irritable bowel syndrome (IBS) and inflammatory bowel diseases (IBD) are gastrointestinal (GI) diseases characterized by abdominal pain and altered bowel patterns. Fatigue and sleep disturbances are prevalent in these GI diseases, with a bidirectional relationship suggested between GI symptoms and sleep quality. If poor sleep results in increased GI symptoms, improving sleep quality may alleviate symptoms. However, if GI symptom burden independently drives poor sleep, alleviating symptoms by disease modification rather than targeting sleep should be the goal of management. Therefore, we aimed to determine if there is a relationship between gastrointestinal symptoms that influences sleep disturbances and/or fatigue.

**Methods:**

A systematic literature search in five databases was conducted using PRISMA guidelines to identify studies addressing fatigue, sleep disturbances, and GI diseases until June 2025. Inclusion criteria were original articles with confirmed GI disease diagnosis and healthy control groups. Data extraction included participant demographics, assessment tools, inflammatory findings, medication use, and disease severity. Quality assessment utilized the Newcastle Ottawa Scale.

**Key Results:**

Of 14,664 articles, 18 studies were included: 7 focused on IBS, 9 on IBD, and 2 on both IBS and IBD. Findings revealed increased fatigue and sleep disturbances in GI patients compared to controls, with IBS patients reporting more fatigue and sleep disturbances than IBD. GI disease severity was strongly associated with sleep quality and fatigue levels.

**Conclusions & Inferences:**

This systematic review highlights the strong association between fatigue and sleep disturbances and GI diseases, which are further exacerbated by disease severity.

## Graphical abstract

Patients with irritable bowel syndrome and inflammatory bowel disease commonly experience poor sleep and fatigue. This is strongly associated with increased GI symptom severity and greatly affects quality of life.

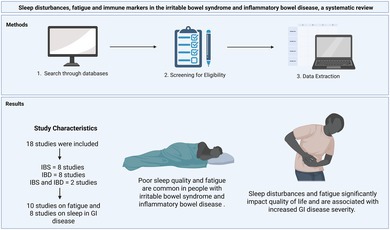

**Summary**

- Poor sleep quality and fatigue are common in people with irritable bowel syndrome (IBS) and inflammatory bowel disease (IBD).
- Sleep disturbances and fatigue are associated with impacts to quality of life and increased GI disease severity.
- IBS patients experience higher levels of sleep disturbance and fatigue compared to IBD patients.

## 1 Introduction

The circadian system plays a crucial role in regulating both the sleep/wake cycle and gastrointestinal (GI) function. This system synchronizes peripheral clocks to the external environment [1], and its disruption can potentially impact both sleep and GI function [2]. Moreover, the circadian system is also involved in modulating the immune system [3, 4, 5] which may have implications for the pathogenesis of GI conditions. There is an increased prevalence of GI symptoms in rotating shift workers [6], and studies have provided strong evidence of a bi‐directional association between GI disease diagnosis and self‐reported poor sleep quality [7, 8]. Additionally, observational studies have suggested that poor sleep quality may be associated with an increased risk or exacerbation of certain GI diseases [9, 10, 11]. Altered sleep patterns are prevalent in patientswith common gastrointestinal (GI) diseases, including the irritable bowel syndrome (IBS) and inflammatory bowel disease (IBD) [6, 12, 13, 14, 15, 16, 17]. Together, this evidence suggests a strong link between sleep disturbances and GI conditions, but whether sleep drives an increased symptom burden or vice versa is unclear.

IBS is a disorder of gut‐brain interaction (DGBI) impacting up to 10% of people globally and is characterized by chronic abdominal pain and alterations in stool consistency [18]. While the etiology of this disease is unknown, there is evidence of intestinal microinflammation in a major subgroup with IBS, including elevated and activated mast cells in the small intestine and colon [19, 20, 21]. Further, there is a high prevalence of fatigue and altered sleep quality in IBS patients [22]. A meta‐analysis found that the prevalence of sleep disorders in IBS is 37.6% [8] and there is strong evidence that sleep disturbances and GI symptoms are strongly linked [23]. Therefore, sleep and fatigue may be important targets in considering the treatment for these patients.

IBD are chronic inflammatory intestinal diseases, with Crohn's disease (CD) and ulcerative colitis (UC) being the two major types. CD is characterized by inflammation anywhere along the GI tract and full thickness inflammation of the affected segments of the intestinal wall, while UC involves inflammation in the mucosa and submucosa of the colon and rectum [24]. Many studies have demonstrated the high prevalence of poor sleep quality and fatigue in IBD patients, with some evidence showing that up to 56% of these patients experience poor sleep [7]. Preclinical studies in murine models have demonstrated the detrimental effect of sleep deprivation on intestinal inflammation and barrier integrity [25, 26, 27]. Further, studies have linked poor sleep quality to an increased risk of developing UC [28] and increased flare incidence in CD [29, 30]. The persistent inflammation and systemic immune activation in these conditions may therefore cause the sleep disturbances and fatigue commonly reported by IBD patients.

Notably, it is unclear whether GI symptoms or other mechanisms (e.g., immune activation) drive sleep and fatigue disturbances, or whether sleep disturbances and fatigue induce GI symptoms, or both pathways are of causal importance. Given the observed relationship between sleep disturbance and inflammation, there is potential for sleep interventions to modulate inflammation and improve barrier integrity [31]. However, the potential peripheral intestinal mechanisms underlying the relationship between sleep disturbances and GI conditions require further exploration.

Understanding the relationship between fatigue, sleep disturbances, and the immune system across these conditions may provide valuable insights into their management and treatment strategies. Therefore, we aimed to investigate the relationship between these factors and determine its relevance to both organic and functional GI diseases.

Our primary research question was ‘Is there a relationship between gastrointestinal disease severity that influences sleep disturbances and/or fatigue?’ We aimed to evaluate the prevalence of sleep disturbances and fatigue in these conditions and explore the role of gastrointestinal symptoms in this context.

## 2 Materials and Methods

### 2.1 Search Strategy

A systematic literature search was conducted in MEDLINE, Embase, Web of Science, Scopus, and Cochrane Library to obtain all publications that reported on fatigue, sleep disturbances, and GI disease published until June 2025. The search strategy included key words: fatigue OR sleepiness OR tiredness OR sleep OR sleep deprivation OR weariness OR sleep disturbance OR night‐time awakening OR insomnia OR sleep disruption combined with gastrointestinal disease OR dyspepsia OR irritable bowel syndrome OR c*eliac disease OR ulcerative colitis OR inflammatory bowel disease OR Crohn Disease OR functional dyspepsia OR DGBI OR Disorder of gut brain interaction OR gluten intolerance OR UC OR IBD OR IBS OR FD OR FGID OR functional gastrointestinal disorder AND immun* OR mechanisms OR inflammation. These terms were searched using limits that included all articles published in the English language. Both text and MeSH terms were searched. PROSPERO systematic review database registration 2022: CRD42022377996. Systematic review with meta analyses that examined associations between fatigue or sleep disturbances in IBS [8, 32] and IBD [7, 32] were hand searched for references.

### 2.2 Study Selection

Three reviewers (SF, LRCD and NS) independently screened titles and abstracts for relevance to the review topic, and the remaining articles underwent full‐text screening for suitability. Inclusion criteria were original articles where sleep disturbances or fatigue and selected GI diseases were studied and used a validated questionnaire to assess sleep disturbances and fatigue. Studies had to have a control group consisting of healthy or general population subjects. Further, IBS had to be confirmed by Rome I–IV criteria and, where undertaken, a normal endoscopy (excluding incidental polyps). Qualitative, quantitative, and mixed methods study designs were included. Organic gastrointestinal disease required clinicopathological diagnosis. Exclusion criteria were studies that looked at cancer or studied pediatric or elderly populations, animal studies, were not in English, conference abstracts, reviews, case studies, and theses (Table 1). Two investigators independently assessed whether articles met the inclusion criteria. Inter‐rater reliability was used to assess agreement between reviewers, which was measured by calculating the percent agreement between reviewers. Conflicts were discussed and resolved between the investigators.

**TABLE 1:** Inclusion and exclusion criteria.

Inclusion criteria	Exclusion criteria
Adults (18–79) Diagnosed coeliac disease, inflammatory bowel disease, irritable bowel syndrome, functional dyspepsia or gluten intolerance (confirmed with endoscopy and Rome criteria for disorders of gut‐brain interaction) Reported fatigue or sleep disturbances (using a validated questionnaire) Full text English Qualitative, quantitative, and mixed methods design. Letters, editorials and trials with appropriate patient data will be eligible for inclusion. Identified control populations and standardized diagnoses	Animal studies Adolescents (under 18 years of age) and elderly people (over 80) Reviews and case studies Cancer related studies

### 2.3 Data Extraction

Studies were grouped based on whether they looked at sleep or fatigue in GI disease. Data extraction included participant demographics, co‐morbidities, fatigue and sleep assessment and criteria used, reported immune findings and measurement details, medication use, gastrointestinal diagnosis and symptoms, and symptom severity where possible. Extraction was performed by two reviewers using a standardized data extraction table for all studies. Conflicts were discussed and resolved between the two investigators.

### 2.4 Quality Assessment

Quality assessment was completed by two independent reviewers using the Newcastle Ottawa Scale [33]. This assesses three domains including the selection of cases and controls, the comparability of cases and controls, and outcome/exposure. Each domain is scored with stars ranging from 0 to 4. The stars are then counted and then converted to Agency for Healthcare Research and Quality (AHRQ) standards [34] and given a rating of good, fair, or poor quality.

## 3 Results

### 3.1 Characteristics of Selected Studies

**FIGURE 1:**
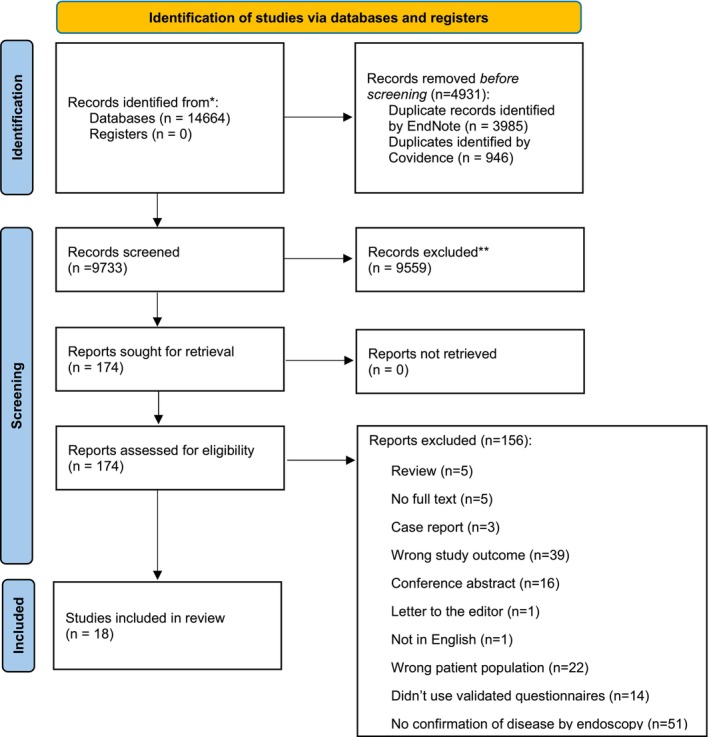
PRISMA flow diagram for the identification and selection of studies. A total of 14664 studies were identified from 5 databases. After removing 4931 duplicate records, 9733 records were screened. Of these, 9559 studies were excluded and 174 papers underwent full‐text screening. A total of 156 studies were excluded and 18 studies were included in the review.

Of the 14,664 articles identified in the search, 4931 were duplicates. Title and abstract screening of 9733 studies resulted in 174 selected for full text review. A total of 156 were then excluded because they were conference abstracts, the wrong patient population, did not use validated questionnaires, or did not have confirmation of disease by endoscopy (Figure 1). The inter‐rater reliability was 98% for title and abstract screening and 55% for full text screening. Eighteen studies were included in the review, which examined cohorts of both IBS and IBD, with eight studies investigating an IBS cohort only, eight had IBD only, and two had IBD and IBS. Further, 10 studies focused on investigating fatigue in IBD and IBS, while 8 studies investigated the role of sleep in GI disease. Reported findings about sleep from studies are summarized in Table 2; reported findings about fatigue are summarized in Table 3. All studies were screened for risk of bias using the Newcastle Ottawa Scale and AHRQ standard. This determined that four studies had poor quality, five had fair quality, and nine had good quality. There was a 100% reviewer consensus on study quality (Table S1). Due to the variability and high heterogeneity between studies, a meta‐analysis could not be performed.

**TABLE 2:** Summary of sleep.

Study	Country	Year	Study design	Sample size	Mean age, *y*	Sex Male, *n* (%)	Disease	Study comparator group	Sample size	Mean age, *y*	Sex male, *n* (%)	Primary outcome/Aim	Sleep measure	Inflammatory marker
Ayadilord et al.	Iran	2020	Case–Control	31	20.4	31 (100)	IBS	No‐IBS symptoms	151	20.7	0 (0)	To investigate whether women who have symptoms of IBS in addition to primary dysmenorrhea and PMS also report more severe or frequent menstruation‐associated symptoms and psychological complications compared to women with primary dysmenorrhea and PMS alone.	ESS, ISS	Not applicable
Balmus et al.	Romania	2020	Case–Control	10	37.57	6 (58)	IBS	Controls	14	39.43	7 (50)	To evaluate several aspects regarding the oxidative stress status in IBS patients' tears. We also aimed to correlate the tears oxidative changes in the context of both IBS symptom severity and sleep disturbances intensity.	PSQI	SOD activity, MDA levels, total soluble proteins levels, glutathione peroxidase activity
Chakradeo at el.	USA	2018	Cross‐Sectional	115	41.4	44 (38)	IBD	Healthy controls	76	34.13	34 (45)	To investigate whether chronotype, social jetlag, sleep debt, and food timing were associated with an IBD specific complications and a lower quality of life.	PSQI	Not applicable
Gilca‐Blanariu et al.	Romania	2020	Case–Control	76	46.2	36 (47)	UC	Healthy controls	66	44.3	26 (40)	To characterize sleep impairment in patients with IBD and to identify potential associated factors	PSQI	Leucocytes, PMN, platelets, CRP, CRP/albumin, fibrinogen, fecal calprotectin
				34	42.6	20 (59)	CD							
Keefer et al.	USA	2006	Case–Control	16	41.44	7 (44)	IBD	Healthy controls	7	34	4 (57)	To precisely characterize the nature of sleep disturbances in patients with IBD and the association of these disturbances with disease outcomes, including quality of life.	PSQI, Epworth Daytime Sleepiness Inventory	Not applicable
				9	52.67	2 (22)	IBS							
Ranjbaran et al.	USA	2007	Case–Control	80	40	33 (41)	IBD	Healthy controls	15	48	7 (47)	To determine whether sleep disturbances are present in IBD and, if so, characterize the nature of this problem in these patients	PSQI	Not applicable
				24	56	7 (30)	IBS							
Wang et al.	China	2023	Case–Control	42	46	9 (21)	IBD	Healthy controls	45	20	16 (35)	To investigate the GABA+ and Glx alterations in the medial prefrontal cortex and its relationship with functional connectivity, as well as its association with brain structural changes in patients with IBD.	PSQI	Not applicable
Zhang et al.	China	2024	Case–Control	208	36.74 (CD) 40.34 (UC)	123 (59)	IBD	Healthy controls	199	36.83	120 (60.3)	To investigate the prevalence and risk factors associated with sleep disorders among patients with IBD	PSQI	Not applicable

Abbreviations: CD, Crohn's disease; CRP, C‐reactive protein SOD, superoxide dismutase; ESR, erythrocyte sedimentation rate; ESS, Epsworth Sleepiness Scale; GABA+, GABA and other neuroactive substances; GLx, glutamate + glutamine; IBD, inflammatory bowel disease; IBS, irritable bowel syndrome; ISS, Insomnia Severity index; MDA, Malondialdehyde; PMN, polymorphonuclear neutrophils; PMS, premenstrual syndrome; PSQI, Pittsburgh Sleep Quality Index; UC, ulcerative colitis.

**TABLE 3:** Summary of fatigue.

Study	Country	Year	Study design	Sample size	Mean age, *y*	Sex Male, *n* (%)	Disease	Study comparator group	Sample size	Mean age, *y*	Sex male, *n* (%)	Primary outcome/Aim	Fatigue measure	Inflammatory marker
Anty et al.	France	2011	Case–Control	42	50.76	8 (19)	IBS	Healthy Controls	44	46.5	21 (48)	To investigate the role of plasma levels of leptin and carnitine on fatigue	FIS	Leptin, total carnitine, free carnitine
Iaquinta et al.	Italy	2022	Cross‐sectional	137	53	126 (61)	IBD	Healthy Controls	206	49	87 (63.5)	To assess the prevalence of fatigue and the associated factors	PROMIS	ESR, CRP
Kvivik et al.	Norway	2021	Case–Control	57	31	22 (39)	CD	Healthy Controls	28	43	5 (18)	To assess the biological basis of fatigue generated by pro‐inflammatory cytokines and other mediators.	VAS	CRP, Fecal calprotectin, anti‐dsHMGB1 antibodies, anti‐frHMGB1 antibodies
Norlin et al.	Sweden	2021	Cross‐Sectional	88	30	13 (15)	IBS	Healthy Controls	47	31	7 (15)	To elucidate fatigue and its association with a resting state network of mesocorticolimbic regions of known importance in fatigue, and to explore the possible role of circulating TNF‐α levels	M‐FIS	TNF‐a
Piche et al.	France	2007	Cross‐Sectional	51	53.7	11 (22)	IBS	Healthy Controls	22	52.3	8 (36)	To evaluate the occurrence of fatigue and its characteristics and to analyze the relationship between fatigue and leptin	VAS	Leptin, adrenocorticotropic hormone, cortisol, thyroid stimulating hormone
Piche et al.	France	2021	Cross‐Sectional	50	53.8	9 (18)	IBS	Healthy Controls	21	56.5	7 (33)	To examine associations between fatigue, depression, and mast cells of the colonic mucosa	FIS	Cellularity of lamina propria, IELs, mast cells, lymphocytes, plasmacytes, eosinophils, neutrophils
Schreiner et al.	Switzerland	2021	Case–Control	1208	49.2	564 (46.5)	IBD	Healthy Controls	414	46.5	181 (40.4)	To identify the prevalence of fatigue in a large IBD cohort compared to the general population, address risk factors, and evaluate its impact on daily life.	VAS, FSS	CRP, fecal calprotectin
Thomann et al.	Germany	2024	Observational Study	67	38.15	23 (34.3)	CD	Healthy Controls	42	38.97	12 (28.5)	To examine gray matter volume alterations in patients with Crohn's disease in different disease states, analyze associations between regions with altered gray matter volumes and symptoms of fatigue, depression, and anxiety, and explore subgroup‐specific characteristics of these associations dependent on disease activity.	WEIMuS	Fecal calprotectin
Tiankanon et al.	Thailand	2021	Cross‐Sectional	209	47.3	103 (49.3)	IBD	Healthy Controls	209	45.1	103 (49)	To compare HRQoL between IBD patients in active disease, clinical remission and steroid‐free clinical remission	FACIT‐F	Not Applicable
Undseth et al.	Norway	2016	Cross‐Sectional	94	40	30 (32)	IBS	Healthy Controls	20	38	8 (40)	To investigate whether fatigue was associated with markers of enterocyte disintegrity, endotoxemia and inflammation.	FIS	iFABP, LPS, soluble CD14, MCP1, calprotectin

Abbreviations: CRP, C‐reactive protein; ESR, erythrocyte sedimentation rate; FACIT‐F, functional assessment of chronic illness therapy – fatigue; FIS, fatigue impact scale; FSS, fatigue severity scale; HMGB1, high mobility group box 1; IBD, inflammatory bowel disease; IBS, irritable bowel syndrome; IELs, intraepithelial lymphocytes; iFABP, intestinal fatty acid binding protein; LPS, lipopolysaccharide; MCP‐1, monocyte chemoattractant protein‐1; PROMIS, patient reported outcomes measurement information system; TNF‐a, tumor necrosis factor alpha; VAS, visual analogue scale; WEIMuS, Würzburg Fatigue Inventory in Multiple Sclerosis scale.

### 3.2 Definitions of Fatigue

Fatigue was evaluated in the studies through a range of validated surveys (Table 4). The Functional Assessment of Chronic Illness therapy—Fatigue (FACIT‐F) survey assesses self‐reported fatigue and the impact on daily activities [35], used in one included study [36]. In contrast, one study [37] used the PROMIS questionnaire to measure self‐reported alertness, sleepiness, tiredness, and functional impairments associated with sleep problems [38]. The Visual Analog Scale (VAS) was utilized by 3 studies [39, 40, 41] and is an 18‐item scale that assesses an individual's subjective experience of fatigue [42]. The Fatigue Severity Scale (FSS), used by one study [41], is a nine‐item questionnaire that measures the impact of fatigue on the individual [43], while three studies used the Fatigue Impact Scale (FIS) [44, 45, 46]. The FIS is 40‐item questionnaire that determines the effect of fatigue on cognitive, physical, and psychosocial functioning [47]. The Modified‐Fatigue Impact Scale (MFIS) reduces the fatigue assessment from 40 to 21 items to assess fatigue [48] and was used in one included study [49]. Another included study [50] used the Würzburg Fatigue Inventory in Multiple Sclerosis (WEIMuS) scale [51] which is a 17‐item questionnaire to assess fatigue. This variability in both definitions and assessment tools makes it challenging to make comparisons across the studies. Further, reporting of results was inconsistent, and variability in reporting of results makes comparisons difficult.

**TABLE 4:** Fatigue comparison.

Study	Country	Year	Study design	Disease	Study comparator group	Fatigue measure	Outcome
Anty et al.	France	2011	Case–Control	IBS	Healthy controls	FIS	Total fatigue score was higher in IBS compared to controls
Iaquinta et al.	Italy	2022	Cross‐sectional	IBD	Healthy controls	PROMIS	Higher incidence of fatigue and higher fatigue score in IBD compared to controls
Kvivik et al.	Norway	2021	Case–Control	CD	Healthy controls	VAS	fVAS score was significantly higher in CD compared to controls
Norlin et al.	Sweden	2021	Cross‐sectional	IBS	Healthy controls	Modified‐FIS	Significantly higher fatigue score in the IBS group compared to controls
Piche et al.	France	2007	Cross‐sectional	IBS	Healthy controls	VAS	IBS group had higher VAS score compared to controls
Piche et al.	France	2021	Cross‐sectional	IBS	Healthy controls	FIS	Higher FIS in IBS group compared to controls
Schreiner et al.	Switzerland	2021	Case–Control	IBD	Healthy controls	VAS, FSS	Patients with IBD reported clinical fatigue significantly more frequently compared to controls and also more severe fatigue
Thomann et al.	Germany	2024	Observational study	CD	Healthy controls	WEIMuS	Patients with CD reported significantly higher fatigue scores. No differences in fatigue scores in active CD compared to remission
Tiankanon et al.	Thailand	2021	Cross‐sectional	IBD	Healthy controls	FACIT‐F	IBD group had significantly increased fatigue compared to controls
Undseth et al.	Norway	2016	Cross‐sectional	IBS	Healthy controls	FIS	No comparison scores between IBS and controls

Abbreviations: FACIT‐F, functional assessment of chronic illness therapy – fatigue; FIS, fatigue impact scale; FSS, fatigue severity scale; IBD, inflammatory bowel disease; IBS, irritable bowel syndrome; PROMIS, patient reported outcomes measurement information system; VAS, visual analogue scale; WEIMuS, Würzburg Fatigue Inventory in Multiple Sclerosis scale.

### 3.3 Fatigue Is Consistently Increased in GI Disease

Regardless of which survey was used, all studies consistently found significantly increased levels of fatigue in individuals with GI disease compared to control groups (Table 4). In IBS, studies using the FIS found that IBS patients experienced more fatigue than controls [44, 45] and this finding was supported by both the M‐FIS [49] and VAS [40] surveys. Anty et al. reported that 55% of IBS patients experienced fatigue, with total fatigue scores significantly higher (68.4 ± 32.9) compared to controls (15.5 ± 11.6) [44]. Similarly, another study found that the M‐FIS scores for IBS were significantly higher (43.0 [32]) than the control group (11.0 [18]), (*p* < 0.001) [49]. Piche et al. found that 62.7% reported fatigue, and controls did not verbally express fatigue. In IBS patients, the total score of fatigue (52.0 ± 30.0) was significantly higher than the control group (21.2 ± 13.6) (*p* < 0.001). Another study found 60% of IBS participants reported fatigue, whilst only one control (4%) did [45]. FIS score was significantly higher in IBS (54 [31–89]) compared to controls (12 [1–19]) (*p* > 0.001). Further, Undesth et al. only reported fatigue scores for the IBS groups (83.4 ± 48.3), precluding direct comparisons with controls [46].

In IBD, fatigue levels were also higher compared to controls and this was a consensus finding across the FACIT‐F [36], PROMIS survey [37], fVAS [39] and FSS [41] surveys. Tiankanon et al. found that IBD patients had a score of 40.9 ± 8.5, while controls scored 45.6 ± 4.3 (*p* < 0.1) on the FACIT‐F [36]. Schreiner et al. reported that 55.6% of IBD patients experienced fatigue, with 11% reporting severe fatigue, compared to 35% of controls who reported fatigue and 3.9% with severe fatigue (*p* < 0.001) [41]. The mean fatigue severity scale scores were 3.2 (1.5) for IBD patients and 3.0 (1.10) for controls (*p* = 0.005). Iaquinta et al. found that fatigue was reported by 34.5% of controls with a median t score of 48 (43‐52), while 54.7% of IBD patients reported fatigue with a median *t*‐score of 52 (46‐50) [37]. Kvivik et al. noted that CD patients had a mean fVAS score of 54.5 (1–98), compared to 14 (2–56) in controls (*p* < 0.001) [39]. Similarly, Thomann et al. found increased fatigue scores in CD (25.00 ± 15.17) compared to controls (8.49 ± 11.80) (*p* < 0.001) [50]. Further, they found that there was no difference in fatigue levels with active CD (28.43 ± 13.92) compared to those in remission (20.52 ± 15.85). This highlights that patients with GI disease have increased fatigue compared to controls.

### 3.4 Definitions of Poor Sleep

Sleep quality was evaluated in the studies through a range of validated surveys (Table 5). The Pittsburgh Sleep Quality Index (PSQI) is a self‐reported questionnaire which assesses sleep quality and disturbances during a 1‐month period [52], used in seven included studies [53, 54, 55, 56, 57, 58, 59] to assess sleep quality. This questionnaire measures subjective sleep quality, sleep latency, sleep duration, habitual sleep efficiency, sleep disturbances, use of sleeping medication, and daytime dysfunction, and gives a score from 0 to 21, with a lower score indicating better sleep. Any score above 5 is defined as poor sleep. One study used the Insomnia Severity Index (ISI) [60], which measures the severity of insomnia, and questions include sleep disorder intensity, sleep‐associated satisfaction, and anxiety‐associated sleep disorder [61]. The total score ranges from 0 to 28, with higher scores indicating more insomnia. Ayadilord et al. [60] did not define insomnia in their study. The Epworth Sleepiness Scale (ESS) measures daytime sleepiness and is measured on a four‐point Likert scale (0–3) with a total score ranging from 0 to 24, and higher scores indicating worse sleepiness [62]. This scale was used in two included studies [59, 60]. Studies by Ayadilord et al. [60] and Keefer et al. [59] did not define what was considered to be worse sleep. The PSQI was the most reported sleep measure which allows for better comparison across studies.

**TABLE 5:** Sleep comparison.

Study	Country	Year	Study design	Disease	Study comparator group	Fatigue measure	Outcome
Ayadilord	2020	Iran	Case–Control	IBS	No‐IBS symptoms	ESS, ISS	IBS group had a worse ISS and ESS score compared to the group with no‐IBS symptoms.
Balmus	2020	Romania	Case–Control	IBS	Controls	PSQI	Increased PSQI score in IBS group as well as IBS‐C and IBS‐D compared to controls.
Chakradeo	2018	USA	Cross‐sectional	IBD	Healthy controls	PSQI	IBD group had higher PSQI scores compared to controls
Gilca‐Blanariu	2020	Romania	Case–control	UC, CD	Healthy controls	PSQI	Higher PSQI score in UC and CD compared to controls.
Keefer	2006	USA	Case–Control	IBD, IBS	Healthy controls	PSQI, Epworth Daytime Sleepiness Inventory	IBD and IBS groups had higher global PSQI scores. IBS group had higher PSQI scores than the IBD group.
Ranjbaran	2007	USA	Case–Control	IBD, IBS	Healthy controls	PSQI	Subjective sleep quality was significantly lower in IBD and IBS compared to controls.
Wang	2023	China	Case–Control	IBD	Healthy controls	PSQI	Higher PSQI score in IBD compared to healthy controls.
Zhang	2024	China	Case–Control	IBD	Healthy Controls	PSQI	Higher PSQI score in both UC and CD compared to healthy controls. No differences between UC and CD.

Abbreviations: CD, Crohn's disease; ESS, Epsworth Sleepiness Scale; IBD, inflammatory bowel disease; IBS, irritable bowel syndrome; ISS, Insomnia Severity index; PSQI, Pittsburgh Sleep Quality Index; UC, ulcerative colitis.

#### 3.4.1 Sleep Quality and Disturbance Are Worse in GI Disease

Regardless of which survey was used, all studies consistently found increased levels of sleep disturbance in individuals with GI disease compared to control groups. In IBS, studies using the ESS, ISS, and PSQI found decreased sleep quality in IBS patients compared to controls (Table 5). Ayadilord et al. [60] discovered that IBS patients reported worse sleep quality and daytime sleepiness compared to non‐IBS patients using the PSQI (5.7 ± 6.4 non‐IBS; 8.7 ± 8.3 IBS; *p* = 0.025), ESS (5.8 ± 5.7 non‐IBS; 8.9 ± 6.2 IBS; *p* = 0.008) and ISI (5.7 ± 6.4 non‐IBS; 8.7 ± 8.3 IBS; *p* = 0.025). Similarly, Balmus et al. [58] found decreased sleep quality in IBS (6.5 ± 1.12) compared to controls (1.79 ± 0.28) (*p* < 0.001), but no differences between IBS subtypes.

This decreased sleep quality was also found in patients with IBD compared to controls across the PSQI, ESS, and ISS surveys. Chakradeo et al. [53] found higher PSQI scores in IBD (6.23 ± 3.5) compared to controls (3.03 ± 1.75; *p* < 0.05). Wang et al. [8] also found this result (3.75 (1.85) control; 6.75 (2.97) IBD; *p* < 0.001). Similarly, another study also found that IBD participants (PSQI = 8) had worse PSQI scores compared to controls (PSQI = 4) (H = 31.3107, *p* < 0.001), but there was no difference between CD and UC patients. Zhang et al. [57] also found this result with both CD (6.71 ± 3.53) and UC (6.90 ± 3.51) patients reporting increased PSQI scores compared to controls (4.78 ± 3.03; *p* < 0.01). This study also reported no differences between CD and UC patients. Further, studies using multiple surveys found consistently poor sleep in IBS and IBD patients compared to the controls, as well as IBS patients reporting worse self‐reported sleep quality compared to IBD patients [55, 59]. Keefer et al. [59] found worse sleep quality in IBS patients (13.25 ± 4.83) compared to controls (6.71 ± 2.23) and IBD patients (9.00 ± 4.84) using the PSQI (*p* < 0.001); however, they observed no differences in daytime sleepiness between any groups using the ESS. This highlights that patients with GI disease consistently report worse sleep quality compared to controls. Ranjbaran et al. [55] found that 71% of IBD and 67% of IBS patients took longer than 30 min to fall asleep, with only 13% of healthy controls reporting this problem; IBD and IBS did not significantly differ, but both differed from controls (*p* < 0.001 IBD vs. controls; *p* = 0.001 IBS vs. controls).

### 3.5 Steroid Use in IBD Patients Is Associated With Increased Fatigue and Sleep Disturbances

Given IBD patients are commonly on medications that alter sleep, we examined whether the studies included accounted for this in their analysis. Of the nine studies focusing on IBD, three factored in the effects of medication on sleep disturbances and fatigue. Iaquinta et al. [37] found that steroid treatment was associated with increased fatigue scores (*p* = 0.014). Similarly, Gilca‐Blanariu et al. [54] reported that IBD patients undergoing corticotherapy had significantly higher PSQI scores (median PSQI score 11; *p* < 0.05) compared to IBD patients that were on other IBD medications, including aminosalicylates, biological therapy, and azathioprine. However, Chakradeo et al. [53] and Zhang et al. [57] found no association between steroid use and increased sleep disturbances as measured by the PSQI.

### 3.6 Anxiety and Depression Is Linked to Increased Fatigue and Worse Sleep Quality in GI Disease

Given mood disorders, such as depression and anxiety, have a strong relationship with fatigue and sleep disturbances, we examined if the studies included accounted for this. Ayadilord et al. [60] used the depression and anxiety scale (DASS)‐21 to compare anxiety and depression levels in IBS compared to non‐IBS patients. IBS patients reported significantly worse depression levels (14.0 ± 10.2) compared to non‐IBS patients (10.5 ± 8.6; *p* = 0.046). No differences were found between groups regarding anxiety levels; however, insomnia was significantly correlated with depression (*p* = 0.003) and anxiety scores (*p* < 0.001).

The hospital anxiety and depression scale (HADS) was utilized by Gilca‐Blanariu et al. [54] which found that the IBD group had higher scores for anxiety (7 (50–10)) compared to controls (4 [2–6]; *p* < 0.0001). IBD patients also had higher levels of depression (8 [5–11]) compared to controls (5 [4–7]; *p* = 0.0063). Furthermore, PSQI scores were significantly correlated with HADS anxiety and depression scores (*p* = 0.0001). Similarly, Ranjbaran et al. [55] found that IBD and IBS patients had higher levels of anxiety compared to controls using the IBD‐Quality of life scale; however, no differences were found between groups regarding depression. Wang et al. [8] used HADS and also found increased depression scores in IBD (16 [7.5–19.5]) compared to controls (5 [2.25–6.00]; *p* < 0.0001). Anxiety levels were also increased in IBD (13.40 ± 3.41) compared to controls (2.88 ± 1.79; *p* < 0.0001). This result was also found by Zhang et al. [57], reporting increased anxiety (controls: 23.1%; CD: 44.7%; UC: 35.4%; *p* < 0.001) and depression (controls: 4.5%; CD: 18.0%; UC: 19.0%; *p* < 0.001) in IBD compared to controls. Anxiety (OR: 2.36; *p* = 0.004) and depression scores (OR: 3.67; *p* = 0.004) were also highly correlated with PSQI scores.

Fatigue was also highly associated with anxiety and depression in both IBD and IBS patients. Iaquinta et al. [37] reported increased anxiety (55 [46–63]) in IBD compared to controls (49 [43–55]; *p* < 0.001). However, there were no differences in depression (controls: 45 [38–51]; IBD 45 [38–56]). Furthermore, there was a significant correlation between fatigue and anxiety (*p* < 0.001) and depression (*p* < 0.001). Similarly, IBD patients with fatigue were reported to have increased anxiety and depression scores using HADS [41]. Thomann et al. [50] observed increased anxiety (controls: 3.43 ± 2.76; IBD: 5.51 ± 3.53; *p* = 0.004) and depression (controls: 1.91 [2.64]; IBD: 3.96 [3.32]; *p* < 0.001) in IBD compared to controls. Furthermore, HADS anxiety and depression scores were found to be significantly correlated with fatigue. Tiankanon et al. [36] also found increased anxiety (IBD: 4.0 ± 4.0; controls: 3.4 ± 2.5; *p* = 0.04) and depression in IBD (IBD: 3.4 ± 3.1; controls: 2.3 ± 2.3; *p* < 0.01) compared to controls. In IBS, HADS anxiety (controls: 3.0 [3.0]; IBS: 10.0 [7.0]; *p* < 0.001) and depression (controls: 1.0 [2.0]; IBS: 5.0 [6.0]; *p* < 0.001) scores were also found to be increased compared to controls [49]. Similarly, Piche et al. [45] used the Beck Depression Inventory (BDI) and reported that 51.5% of IBS patients had a score lower than 4, indicating depression. Furthermore, BDI score was higher in IBS compared to controls (*p* < 0.001) and fatigue score was strongly associated with BDI score (*r* = 0.57, *p* = 0.04). Collectively, this data highlights that anxiety and depression symptoms are strongly associated with increased fatigue and sleep disturbances.

#### 3.6.1 GI Symptom Severity Is Associated With Increased Fatigue and Worse Sleep Quality

There is a correlation between GI symptoms and associations with fatigue and sleep quality (Table 6); however, only 5 out of the 17 papers reported associations with specific GI symptoms. Balmus et al. [58] observed that IBS patients experienced increased abdominal pain intensity (4:1 ± 0:65) compared to controls (1:428 ± 0:17) (*p* < 0.001). Further, IBS patients exhibited increased PSQI scores, indicative of poorer sleep quality, and slept fewer total hours than the control group. Piche et al. [45] observed that IBS patients reported a higher severity of abdominal pain and discomfort (2.5 [0.9]) compared to controls (0.1[0.4]) (*p* = 0.001), along with worse abdominal well‐being (IBS: 5.7 [1.6]; controls 0.6 [0.5]; *p* = 0.001). Interestingly, there were no differences observed between IBS subtypes. However, the severity of abdominal pain and discomfort and the general abdominal well‐being of IBS patients were not associated with fatigue scores. In contrast, Norlin et al. found that fatigue was related to IBS symptom severity (IBS: *r* = 0.36; controls: *r* = 0.26) [49].

**TABLE 6:** Summary of findings: GI symptoms.

Study/Year	Sample size	Disease	Assessment method	GIT symptom assessment	Outcomes
Balmus et al. (2020)	*n* = 14 controls, *n* = 10 IBS	IBS	PSQI	VAS–IBS	IBS group had higher VAS‐IBS scores, abdominal pain, diarrhea, constipation and bloating/gases compared to controls
Chakradeo et al. (2018)	*n* = 76 controls, *n* = 115 IBD	IBD	PSQI	SIBDQ, CDAI, modified Harvey‐Bradshaw questionnaire	IBD subjects with higher PSQI scores had lower SIBDQ scores Increased disease activity was associated with worse subjective sleep quality, increased sleep onset latency, and increased daytime dysfunction
Gilca‐Blanariu et al. (2020)	*n* = 66 controls, *n* = 76 UC, *n* = 34 CD	IBD	PSQI	Abdominal pain, Mayo score	Increased abdominal pain in UC compared to CD and control group Active disease reported higher PSQI scores PSQI score was correlated with Mayo score
Iaquinta et al. (2022)	*n* = 26 controls, *n* = 137 IBD	IBD	PROMIS	Harvey Bradshaw Index, Mayo Score	Disease activity was significantly correlated with fatigue scores
Keefer et al. (2006)	*n* = 7 controls, *n* = 16 IBD, *n* = 9 IBS	IBD	PSQI	IBDQ	PSQI score was correlated with disease‐specific quality of life
Kvivik et al. (2021)	*n* = 28 controls, *n* = 57 CD	CD	Modified‐FIS	Simple endoscopic score for Crohn's disease	No correlation between simple endoscopic score and fatigue levels
Norlin et al. (2021)	*n* = 47 controls, *n* = 88 IBS	IBS	Modified‐FIS	IBS‐SSS	Fatigue was related IBS‐SSS score
Piche et al. (2008)	*n* = 21 controls, *n* = 50 IBS	IBS	FIS	Severity of abdominal pain/discomfort	Higher severity of abdominal pain and discomfort in IBS group compared to controls but not associated with fatigue scores
Ranjbaran et al. (2007)	*n* = 18 controls, 80 = IBD, *n* = 24 IBS	IBD	PSQI	IBD‐Questionnaire, severity of abdominal pain	Subjects with more severe disease symptoms had worse sleep quality Severity of abdominal pain and cramps were significantly associated with poorer reported sleep quality in IBD but not in IBS
Schreiner et al. (2021)	*n* = 414 controls, *n* = 1208 IBD	IBD	VAS, FSS	Nocturnal Diarrhea	More nocturnal diarrhea in IBD patients that experience fatigue compared to IBD patients that didn't experience fatigue Patients with fatigue had higher CDAI scores
Tiankanon et al. (2021)	*n* = 209 controls, *n* = 209 IBD	IBD	FACIT‐F	SIBDQ	Patients with active disease had a lower FACIT fatigue score

Abbreviations: CD, Crohn's disease; CDAI, Crohn's disease activity index; FACIT‐F, functional assessment of chronic illness therapy—fatigue; FIS, fatigue impact scale; FSS, fatigue severity scale; IBD, inflammatory bowel disease; IBDQ, IBD quality of life; IBS, irritable bowel syndrome; PSQI, Pittsburgh Sleep Quality Index; UC, ulcerative colitis; VAS, visual analogue scale; VAS‐IBS, visual analogue scale‐irritable bowel syndrome.

Gilc‐Blanariue et al. [54] found increased abdominal pain in UC compared to CD and controls, and IBS and IBD patients collectively displayed higher PSQI scores than controls, with disease activity significantly impacting sleep quality. Interestingly, patients with active disease reported higher PSQI scores (9 [6–12]) than those in remission (7 [4–9]), and a correlation between PSQI score and Mayo score was identified (*r* = 0.41; *p* < 0.0001). Chakradeo et al. [53] found that IBD subjects with higher PSQI scores had lower SIBDQ scores (*r* = −0.445, *p* < 0.05). Further, increased disease activity was associated with worse subjective sleep quality (*r* = −0.503, *p* < 0.05), increased sleep onset latency (*r* = −0.285, *p* < 0.05), and increased daytime dysfunction (*r* = −0.539, *p* < 0.05). Using regression analysis, Keefer et al. [59] found that the PSQI global score was significantly correlated with disease‐specific quality of life (*r* ^2^ = 0.63, *p* = 0.001). Schreiner et al. [41] found that IBD patients with fatigue experienced more nocturnal diarrhea (23.3%) compared to those without fatigue (7.5%). Patients with fatigue had higher scores for the Crohn's Disease Activity Index (37 [42.5]) than IBD patients without fatigue (24.1 [28.5]) (*p* < 0.0001). Iaquinta et al. [37] found that disease activity was correlated with fatigue scores (odds ratio 2.12, 95% CI [1.13–3.97]; *p* = 0.019). Tiankanon et al. [36] found that patients with active disease had a lower FACIT fatigue score (39.2 ± 9.4) compared to those in clinical remission (42.5 ± 7.2). In contrast, Kvivik et al. [39] found no correlation between simple endoscopic score and fatigue levels.

Ranjbaran et al. [55] found that the IBD‐Questionnaire score inversely correlated with sleep quality reported on the PSQI (*r* ^2^ = 0.55, *p* = 0.02) such that subjects with more severe disease symptoms had worse sleep quality. Similarly, the severity of abdominal pain (*p* = 0.04) and cramps (*p* = 0.01) were significantly associated with poorer reported sleep quality in IBD but not in IBS.

Collectively, these studies support a connection between GI symptoms and the experience of fatigue and sleep quality among patients, potentially influencing the severity of the underlying disease.

### 3.7 Immune Activation Is a Risk Factor Associated With Sleep Disturbances and Fatigue

In these studies, a wide range of biomarkers and parameters was investigated to explore inflammation in GI disease. However, only a limited number of studies examined these immune markers in the context of reported fatigue and/or sleep disturbance. Two out of seven studies looking at sleep disturbances and nine out of ten studies on fatigue included data on immune markers (Table 7).

**TABLE 7:** Summary of findings: Inflammation.

Study/Year	Sample size	Disease	Fatigue assessment method	Inflammatory markers analysis	Outcomes
Anty et al. (2011)	*n* = 44 controls, *n* = 42 IBS	IBS	French‐FIS	Leptin, total carnitine, free carnitine	IBS group had increased leptin compared to controls Total fatigue score was significantly correlated with leptin
Balmus et al. (2020)	*n* = 14 controls, *n* = 10 IBS	IBS	PSQI	SOD activity, MDA levels, total soluble proteins levels, glutathione peroxidase activity	IBS group had increased SOD, MDA and total soluble protein levels compared to controls
Gilca‐Blanariu et al. (2020)	*n* = 66 controls, *n* = 76 UC, *n* = 34 CD	IBD	PSQI	CRP, CRP/albumin, fibrinogen, fecal calprotectin	UC group had a higher fecal calprotectin compared to CD Correlation between CRP, fibrinogen and calprotectin and PSQI scores in the IBD group
Iaquinta et al. (2022)	*n* = 206 controls, *n* = 137 IBD	IBD	PROMIS	ESR, CRP	No differences in inflammatory markers
Kvivik et al. (2021)	*n* = 28 healthy controls, *n* = 57 CD	CD	fVAS	CRP, Fecal calprotectin, anti‐dsHMGB1 antibodies, anti‐frHMGB1 antibodies	No differences were found in antibody levels
Norlin et al. (2021)	*n* = 47 controls, *n* = 88 IBS	IBS	Modified fatigue impact scale	TNF‐a	IBS groups had significantly higher plasma levels of TNF‐α compared to controls
Piche et al. (2007)	*n* = 22 controls, *n* = 51 IBS	IBS	VAS	Leptin, adrenocorticotropic hormone, cortisol, thyroid stimulating hormone	IBS group had significantly higher leptin levels compared to controls
Piche et al. (2008)	*n* = 21 controls, *n* = 50 IBS	IBS	FIS	Cellularity of lamina propria, IELs, mast cells, lymphocytes, plasmacytes, eosinophils, neutrophils	IBS group had higher cellularity of lamina propria, mast cells and lymphocytes Cellularity of the lamina propria and mast cells was correlated with FIS score in IBS
Schreiner et al. (2021)	*n* = 414 controls, *n* = 1208 IBD	IBD	VAS, FSS	CRP, fecal calprotectin	No differences found in CRP and fecal calprotectin levels
Thomann et al.	*n* = 42 controls, *n* = 67 CD	CD	WEIMuS	Fecal calprotectin	WEIMuS and fecal calprotectin levels negatively correlated in CD patients in remission and were positively associated in active CD
Undseth et al. (2016)	*n* = 20 healthy controls, *n* = 94 IBS	IBS	FIS	iFABP, LPS, soluble CD14, MCP1, calprotectin	Controls had a higher iFABP score compared to IBS group

Abbreviations: CD, Crohn's disease; CRP, C‐reactive protein; ESR, erythrocyte sedimentation rate; ESS, Epsworth Sleepiness Scale; FACIT‐F, functional assessment of chronic illness therapy – fatigue; FIS, fatigue impact scale; FSS, fatigue severity scale; FSS‐fatigue severity scale; HMGB1, high mobility group box 1; IBD, inflammatory bowel disease; IBS, irritable bowel syndrome; IELs, intraepithelial lymphocytes; iFABP, intestinal fatty acid binding protein; ISS, Insomnia Severity index; LPS, lipopolysaccharide; MCP‐1, monocyte chemoattractant protein‐1; MDA, Malondialdehyde; PMN, polymorphonuclear neutrophils; PROMIS, patient reported outcomes measurement information system; PSQI, Pittsburgh Sleep Quality Index; SOD, superoxide dismutase; TNF‐a, tumor necrosis factor alpha; UC, ulcerative colitis; VAS, visual analogue scale; WEIMuS, Würzburg Fatigue Inventory in Multiple Sclerosis scale.

#### 3.7.1 Blood Cytokines and Immune Markers

Norlin et al. [49] found higher tumor necrosis factor‐alpha (TNF‐α) levels in plasma in IBS patients. TNF‐α levels were also positively correlated with fatigue in IBS patients (*r* = 0.28, *p* = 0.001) but not in controls (*r* = 0.13, *p* = 0.37). Undseth et al. [46] measured intestinal fatty acid‐binding protein (iFABP), monocyte chemoattractant protein‐1 (MCP‐1) and calprotectin in serum in IBS; lipopolysaccharide (LPS) and soluble cluster of differentiation 14 (sCD14) were measured in plasma. The results showed a higher iFABP score in controls compared to individuals with IBS. Further, elevated levels of anti‐HMGBi auto‐antibodies were associated with reduced fatigue in individuals with Crohn's disease. However, none of these parameters were correlated with fatigue scores. Kvivik et al. [39] measured anti‐double‐stranded high‐mobility group box 1 (Anti‐dsHMGB1) antibodies and anti‐frizzled high‐mobility group box 1 (Anti‐frHMGB1) antibodies in plasma. No differences were found between CD and healthy controls in antibody levels, and no significant correlations were found between serum calprotectin levels and fatigue.

#### 3.7.2 IBS Is Associated With Increased Leptin Levels and Fatigue

Anty et al. [44] investigated leptin levels in serum, as well as total carnitine and free carnitine in plasma as they play a role in energy metabolism. They found increased levels of leptin in the IBS group compared to controls, and the total fatigue score was significantly correlated with leptin (*r* = 0.31, *p* = 0.04). Similarly, Piche et al. [40] measured leptin, adrenocorticotropic hormone (ACTH), cortisol, and thyroid stimulating hormone (TSH). Interestingly, they also observed increased leptin levels in the IBS group as well as a positive association between total fatigue score and leptin level (*r* = 0.49; *p* = 0.0003).

#### 3.7.3 Colonic Markers and Sleep/Fatigue

Piche et al. [45] investigated the cellularity of the colonic lamina propria, including intraepithelial lymphocytes (IELs), mast cells, lymphocytes, plasmacytes, eosinophils, and neutrophils, using biopsies from the caecum and terminal ileum. The findings indicated higher cellularity in the lamina propria, particularly in mast cells and lymphocytes, in individuals with IBS compared to controls. Further, the cellularity of the lamina propria (*r* = 0.51, *p* = 0.0001) and the mast cells (*r* = 0.64, p,0.0001) was significantly correlated with FIS score in the IBS group. In IBD, Gilca‐Blanariu et al. [54] found higher levels of fecal calprotectin in UC compared to CD. Further, there was a significant correlation between c‐reactive protein (CRP), fibrinogen, and calprotectin and PSQI scores in the IBD group (CRP: *r* = 0.22, *p* = 0.0013; fibrinogen: *r* = 0.21, *p* = 0.0029; calprotectin: *r* = 0.48, *p* = 0.036). Moreover, the neutrophil to lymphocyte ratio (*r* = 0.21, *p* = 0.0280) and CRP to albumin ratios (*r* = 0.32, *p* = 0.001) were also significantly correlated with PSQI score. In CD, Thomann et al. [50] found that patients in remission demonstrated a negative correlation between fatigue scores and fecal calprotectin (*r* = −0.4) whilst fatigue scores in those with active CD were positively correlated with fecal calprotectin (*r* = 0.44). In contrast, Kvivik et al. [39] found no significant correlations with fatigue and calprotectin levels in IBD. Similarly, Schreiner et al. [41] also did not find any significant correlations with fatigue and calprotectin levels in IBD.

## 4 Discussion

The main findings of this systematic review support the notion of elevated sleep disturbance and fatigue in IBS and IBD. Further, GI symptom severity and disease activity are strongly associated with sleep quality and fatigue levels. Self‐reported findings indicate worse sleep quality in IBS compared to IBD patients; however, this is not confirmed in polysomnography (PSG) data [32, 63]. A study by Heitkamper et al. found increased self‐reported sleep disturbances in women with IBS, and that there was a weak relationship between self‐reported and PSG data [64, 65]. This highlights that perception of fatigue may be altered in IBS. The literature shows that IBS patients have an altered pain perception and increased sensitivity [66], including alterations in central pain processing and dysfunction in inhibitory pain systems [67]. In addition, patients with IBS exhibit exaggerated symptom burdens when assessed by recall, compared to when symptoms are prospectively recorded [68]. Therefore, IBS patients' perception of sleep quality is altered too, or their ability to tolerate poor sleep may be reduced, resulting in increased perception of fatigue. However, the direction of these pathways is not well understood, and further studies are needed to fully elucidate this mechanism.

There is evidence of a strong correlation between GI disease severity and worse sleep quality and fatigue [69, 70]. Our findings further emphasize this result, highlighting that disease activity and GI symptoms were associated with poor sleep and a high prevalence of fatigue in multiple studies across IBS and IBD. However, some studies found no correlation between GI symptoms and fatigue and sleep quality [39, 45]. One study found that IBD disease severity was associated with poor sleep; however, this was not found in the IBS group [55]. This may be related to the levels of inflammation, as IBD is associated with pathological inflammatory profiles compared to IBS, which may cause worse sleep and fatigue. Studies have shown that inducing inflammation leads to altered sleep [71, 72]. Il‐1β and TNF‐ α are involved in the regulation of sleep, specifically the promotion of non‐rapid eye movement sleep [73]. Further, high levels of these cytokines are associated with feelings of sleepiness and fatigue [74]. The literature also highlights that poor sleep can result in increased inflammation, which could also further exacerbate these conditions [26]. The exact pathway of this is unclear, though, and further research is necessary to fully elucidate these mechanisms.

Two studies identified elevated levels of leptin in IBS [40, 44], indicating a potential involvement of this hormone in IBS pathophysiology. Leptin regulates energy balance, metabolism, and body weight [75, 76] through leptin receptor isoform b neurons, primarily located in the hypothalamus [77]. Of importance here, signaling of leptin may be influenced by the SCN, which controls sleep cycles [78, 79]. As such, plasma leptin levels show circadian rhythmicity, with levels increasing during the day and peaking at night [80], a similar rhythm to the hormone melatonin. Further, sleep restriction has been shown to alter the secretion of leptin [81, 82], highlighting the influence of the circadian system on this hormone. Leptin has been associated with fatigue in chronic fatigue syndrome and chronic hepatitis C [83, 84]. Leptin can also modulate innate inflammatory responses, including cytokine production from macrophages and mast cells [85], induce IL‐6 and TNF [86] and promote NK activation [87]. Consequently, leptin might contribute to immune activation in IBS via its role in inhibiting regulatory T cells [88], but further investigations are necessary to explore this connection.

There were several limitations in this study. First, there was high heterogeneity between studies in terms of gender, age, settings, and study design. Due to the design of the studies included, the findings are all observational, so causality cannot be established, which limits the ability of these studies to understand the true relationship between sleep and fatigue on IBS and IBD. Additionally, many studies utilized different fatigue and sleep measurement tools, making comparisons challenging and potentially biasing the estimation of fatigue and sleep disturbances in the cohorts. These limitations also meant we could not perform a meta‐analysis. Relatively few studies investigated potential organic causes of fatigue, limiting our ability to rule out underlying physiological explanations. No studies included in our systematic search reported measures of both sleep and fatigue in the same cohort. This could provide further information on how sleep and fatigue affect daily functioning; impaired quality of life and altered pain perception may contribute to increased levels of fatigue in patients with IBS. Further, six of the studies looking at IBD cohorts did not account for medication usage, which could impact fatigue and sleep disturbances. We had originally planned to include coeliac disease in this review, but we found only one study that focused on this, so we were unable to obtain an understanding of the relationship between sleep patterns and fatigue in patients with coeliac disease. In the cohorts examined, there was no subtyping of IBS patients and subsequent characterization of their fatigue and sleep profiles, so the exact role of sleep and fatigue between different subtypes was unable to be determined. Patients with IBD and IBS reported increased abdominal pain compared to controls, which is unsurprising; however, no studies looked at differences between different GI diseases, which is a limitation of the literature.

These studies collectively highlight the complex relationship between immune system function, sleep disturbances, and fatigue in IBS and IBD. While some immune markers and factors such as leptin have been associated with fatigue in IBS, the exact mechanisms remain to be fully understood, and there is a need to investigate their role in fatigue among IBS and IBD patients. Further, disease activity and severity are strongly associated with worse sleep quality and fatigue, so targeting this may help reduce GI symptoms. However, the question of whether sleep disturbances and fatigue drive increased symptom burden or if symptom burden results in increased fatigue and sleep disturbances still remains unclear. Further research is needed to ascertain the specific roles of sleep and fatigue in chronic GI diseases.

## Author Contributions

S.F., L.R.C.D., and N.S. performed a systematic search of the literature. S.F. and G.L.B. wrote the manuscript. S.K., N.J.T., and G.L.B. reviewed the manuscript. All authors reviewed and approved the final manuscript.

## Conflicts of Interest

S.F., LRCD, N.S. declare no conflicts of interest. NJT: Non‐financial support from: Norgine (2021) (IBS interest group), personal fees from Allakos (gastroduodenal eosinophilic disease) (2021), Bayer (IBS) (2020), Planet Innovation (Gas capsule IBS) (2020), twoXAR Viscera Labs, (USA 2021) (IBS‐diarrhea), Dr. Falk Pharma (2020) (EoE), Sanofi‐aventis, Glutagen (2020) (celiac disease), IsoThrive (2021) (esophageal microbiome), BluMaiden (microbiome advisory board) (2021), Rose Pharma (IBS) (2021), Intrinsic Medicine (2022) (human milk oligosaccharide), Comvita Mānuka Honey (2021) (digestive health), Astra Zeneca (2022), outside the submitted work. In addition, Dr. Talley has a patent Nepean Dyspepsia Index (NDI) 1998, Biomarkers of IBS licensed, a patent Licensing Questionnaires Talley Bowel Disease Questionnaire licensed to Mayo/Talley, a patent Nestec European Patent licensed, and a patent Singapore Provisional Patent “Microbiota Modulation of BDNF Tissue Repair Pathway” issued, “Diagnostic marker for functional gastrointestinal disorders” Australian Patent Application WO2022256861A1via the University of Newcastle and UniQuest (University of Queensland). Dr. Talley is supported by funding from the National Health and Medical Research Council (NHMRC) to the Centre for Research Excellence in Digestive Health and he holds an NHMRC Investigator grant. GLB: Diagnostic marker for functional gastrointestinal disorder' Australian Patent Application WO2022256861A1via the University of Newcastle and UniQuest (University of Queensland). SK: Grants from National Health and Medical Research Council (Ideas Grant and Centre for Research Excellence), grants from Viscera Labs (Research contract), grants from Microba Life Science (Research contract), personal fees from Gossamer Bio, personal fees from Anatara Lifescience, personal fees from Immuron, personal fees from Microba Life Science. Diagnostic marker for functional gastrointestinal disorder' Australian Patent Application WO2022256861A1via the University of Newcastle and UniQuest (University of Queensland).

## Supporting information


**Table S1.** Quality scoring.

## Data Availability

Data sharing not applicable to this article as no datasets were generated or analysed during the current study.
