# Supplementary material for: Sleep Disturbances, Fatigue and Immune Markers in the Irritable Bowel Syndrome and Inflammatory Bowel Disease, a Systematic Review
Source: Neurogastroenterol Motil. 2025 Aug 31;37(11):e70133. doi: 10.1111/nmo.70133 (PMC12534578; doi:10.1111/nmo.70133)
Supplement: Supplementary file 1 — Table S1. Quality scoring. [file NMO-37-e70133-s001.pdf]

Supplementary Table 1. Quality Scoring

Study	NOS Selection	NOS Comparability	NOS Outcome	NOS Score	AHRQ Score
Anty et al. 2011	4	2	2	8/9	Good
Ayadilord et al. 2020	1	0	2	3/9	Poor
Balmus et al. 2020	3	2	2	7/9	Good
Chakradeo et al. 2018	2	1	2	5/9	Fair
Gilca-Blanariu et al. 2020	2	2	2	5/9	Fair
Iaqunito eta l. 2022	3	2	2	7/9	Good
Keefer et al. 2006	3	1	2	6/9	Good
Kvivik et al. 2021	2	2	2	6/9	Fair
Norlin et al. 2021	3	2	2	7/9	Good
Piche et al. 2007	3	1	2	6/9	Good
Piche et al. 2008	2	1	2	5/9	Fair
Ranjbaran et al. 2007	2	0	2	3/9	Poor
Schreiner et al. 2021	4	2	2	8/9	Good
Thomann et al. 2024	2	2	2	6/9	Good
Tiankanon et al. 2021	3	1	2	6/9	Good
Undseth et al. 2016	1	0	1	2/9	Poor
Wang et al. 2023	0	1	2	3/9	Poor
Zhang et al. 2024	1	2	2	5/9	Fair

AHRQ=Agency for Healthcare Research and Quality. NOS= Newcastle Ottawa scale.
